# Personalized resilience: how individual variability in brain–immune responses to stress influences the development of anxiety disorders

**DOI:** 10.3389/fncel.2026.1745994

**Published:** 2026-03-03

**Authors:** Nadine Peixoto, Rosalina Fonseca

**Affiliations:** Learning and Plasticity, i3s - Instituto de Investigação e Inovação em Saúde, Universidade do Porto, Porto, Portugal

**Keywords:** stress, resilience, amygdala activation, endocannabinoid system, cortisol, cytokines

## Abstract

Stress exposure has increased significantly, contributing to higher rates of anxiety, depression, and post-traumatic stress disorder. However, individual responses to stress vary greatly, underscoring the concept of stress resilience. The acute stress response activates the amygdala, hippocampus, and prefrontal cortex, stimulating the HPA axis and triggering cortisol release, which typically restores balance through negative feedback mechanisms. In contrast, chronic stress or exposure to severe stressors leads to sustained HPA axis activation, amygdala hyperreactivity, and immune dysfunction, all of which promote the development of anxiety disorders. This review explores potential central and peripheral biomarkers of resilience, emphasizing the interplay between immune responses, the HPA axis, and the endocannabinoid signaling system. We discuss whether amygdala reactivity could serve as a predictor of stress vulnerability, along with cortisol levels and sleep disturbances, as these are often hallmarks of stress-related disorders. Furthermore, we suggest that pro-inflammatory cytokines such as TNF-α and IL-6–key indicators of stress-induced inflammation–may serve as predictors of anxiety-related vulnerability. Furthermore, we discuss the potential role of the endocannabinoid system as an integrative hub for stress responses. Given its capacity to coordinate central and peripheral mechanisms–from neuroimmune to metabolic processes–we examine how genetic and functional variations in CB1R and FAAH may influence individual resilience, highlighting their potential as biomarkers of stress susceptibility. Clinically is of utmost relevance the identification of reliable and reproducible biomarkers for advancing diagnostic precision and developing personalized therapeutic interventions for stress-related disorders.

## Introduction

In contemporary society, exposure to stress has unfortunately become a common experience. As chronic stress has increased substantially, so has the incidence of stress-related disorders, including generalized anxiety disorder (GAD), depression, and post-traumatic stress disorder (PTSD) (Zhai and Du, 2024; Haller et al., 2014). It is important to note that according to the DSM-5-TR (Diagnostic and Statistical Manual of Mental Disorders, 5th Edition, Text Revision), PTSD and anxiety disorders, such as GAD, are classified into separate categories. This is mainly based on the necessity of an identifiable trauma for PTSD diagnosis and not because the physiological mechanisms underlying PTSD and GAD development and clinical presentation are significantly different (Bandelow et al., 2017; Daviu et al., 2019). Importantly, individual responses to stress are far from uniform; some individuals exhibit remarkable psychological adaptability and stability when confronted with similar stressors (Fonseca et al., 2021). This variability in stress responses has led to a growing interest in the concept of resilience, defined as the ability to maintain or regain mental health despite adversity. Individual differences in genetic, epigenetic, developmental, psychological, and neurochemical factors play a significant role in determining subject resilience and, therefore, understanding why some individuals are more resilient than others has become a key question toward the development of a personalized approach to stress-related brain disorders (Wu et al., 2013). In this review, we aim at exploring possible central and peripheral biomarkers of stress resilience, focusing on the relationship between immune responses and cortisol dynamics and the endocannabinoid signaling system.

## Individual factors affecting stress resilience

**FIGURE 1:**
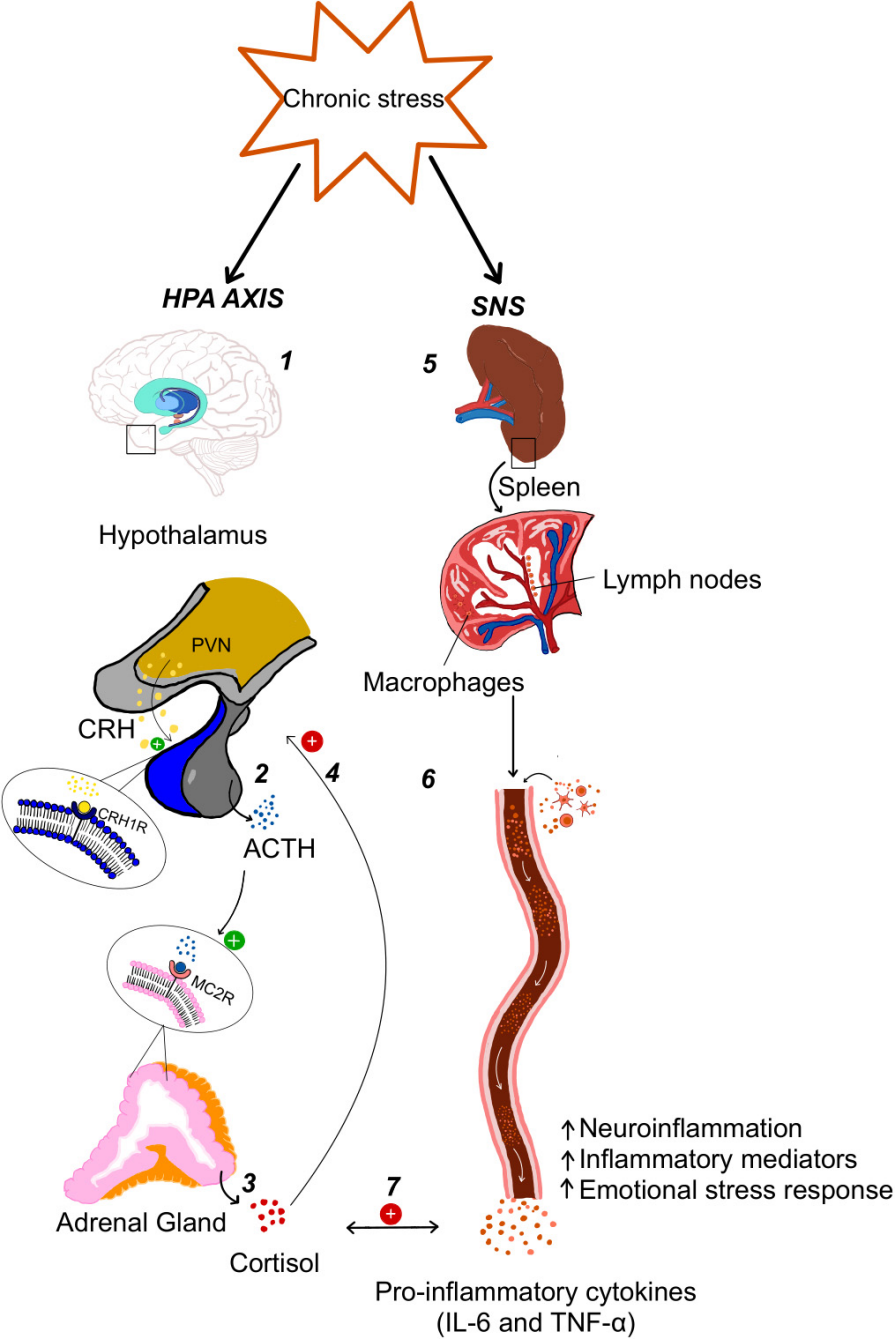
The activation of the Hypothalamic-Pituitary-Adrenal (HPA) axis and Sympathetic Nervous System (SNS) in chronic Stress. **1**: In the hypothalamus, neurons from the paraventricular nucleus (PVN) synthesize and release corticotropin-releasing hormone (CRH), which **2**: stimulates the anterior pituitary to secrete ACTH (adrenocorticotropic hormone) by binding to its receptor (CRH1R); **3**: ACTH, in turn, acts on the adrenal cortex, through the MC2 receptor leading to cortisol release. **4**: The negative feedback that cortisol normally exerts on the axis is impaired in chronic stress leading to the opposite effect. **5**: Sympathetic activation affects the spleen and other immune organs where macrophages and lymph nodes are stimulated **6**: leading to the release of pro-inflammatory cytokines. These increase neuroinflammation, systemic inflammation and emotional stress response. **7**: Both cortisol and cytokines can influence each other in a bidirectional feedback loop promoting stress-related immune dysregulation.

Stressors can be defined as any internal or external event, condition, or stimulus that challenges an individual’s ability to cope, adapt, or maintain psychological or physiological equilibrium, thereby triggering a stress response, with individual perception playing a crucial role in determining their impact (Wheaton, 1999). The effect of a stressor on an individual largely depends on several personal factors, such as one’s physical state, broader social context, sex and coping strategies. Moreover, different types of stressors can affect mental health in different ways and have cumulative effects, increasing the risk of depression and anxiety, compromising well-being (Al Huwailah et al., 2023; McLoughlin et al., 2021). Given all these confounding factors, identifying individual factors that may guide clinicians to determine prognosis and possible clinical interventions is critical. Acute stress typically occurs in response to a specific event and is temporary, typical involving the activation of the amygdala, hippocampus, and prefrontal cortex, key brain structures responsible for regulating emotional responses to fear. Activation of the paraventricular nucleus (PVN) of the hypothalamus, by the amygdala or brainstem nuclei, leads to the release of corticotropin-releasing hormone (CRH). CRH then acts on the anterior pituitary gland to promote the secretion of adrenocorticotropic hormone (ACTH). In turn, ACTH stimulates the adrenal cortex to release glucocorticoids, primarily cortisol in humans (or corticosterone in rodents). Through binding to intracellular glucocorticoid receptors (GRs) (Figure 1), these hormones mediate a wide range of metabolic, immunologic, and behavioral adaptations to stress (Herman et al., 2005). This physiological stress response is adaptive and is terminated by feedback mechanisms involving cortisol and a negative modulation of PVN, hippocampus, amygdala and prefrontal cortex contributing to the termination of the hypothalamic-pituitary-adrenal axis (HPA) response (de Kloet, 2000; Dallman, 2005; Ulrich-Lai and Herman, 2009). On the other hand, chronic stress involves prolonged exposure to stressors without adequate recovery time (Wheaton, 1997), leading to a constant activation of all structures involved in the stress response, particularly the HPA axis (Mizoguchi et al., 2003). This hyperactivation of the amygdala and prolonged cortisol release leads to the desensitization of negative feedback mechanisms, triggering a dysfunctional neuroendocrine status (Zhang et al., 2019). This unbalanced chronic amygdala reactivity results in altered patterns of connectivity with the ventromedial prefrontal cortex (vmPFC) and the hippocampus, altering sleep patterns and memory formation (Porta-Casteràs et al., 2020). Although other systems are also altered by chronic stress, can baseline reactivity of the amygdala be predictive of whether individuals are resilient or not to stress? Indeed, several imaging studies, using functional resonance imaging (fMRI), have shown that the degree of basal amygdala reactivity to negative stimuli is a risk factor for the development of mood disorders in the context of prolonged exposure to stress (Bürger et al., 2023; Blasi et al., 2009; Mattson et al., 2016). However, the technical complexity involved in collecting and analyzing amygdala responsiveness of patients renders this parameter inadequate as a clinical biomarker.

Can cortisol levels be used as a predictor of susceptibility to stress disorders? The use of cortisol as a biomarker for PTSD remains controversial, as findings across studies are inconsistent. The heterogeneous results reported across studies assessing cortisol levels in PTSD can be attributed to several methodological and biological factors. These include variations in sampling protocols (e.g., assessment of basal versus stress-induced levels), differences in the biological source of cortisol measurement (such as plasma, saliva, urine), sex-specific effects, and the time passed since trauma exposure (Lawrence and Scofield, 2024; Sbisa et al., 2023). While some studies have reported elevated cortisol levels in PTSD patients (Baker et al., 2005; Carrion et al., 2002; Song et al., 2008), others have described reduced concentrations (Pan et al., 2018, 2020). Furthermore, Engel et al. (2023) showed that experimental trauma exposure induces a cortisol response that does not consistently predict PTSD symptoms. Given these discrepancies, cortisol levels are not a good biomarker of disease severity, but can acute cortisol release be a biomarker of susceptibility/resilience? As stated above, acute cortisol release upon stress exposure is protective, and indeed, a higher anticipatory cortisol release to acute stress was associated with better mental health outcomes following major life stress (Shao et al., 2023). A similar result was seen when correlating cortisol levels and treatment outcome, suggesting that higher reactivity is a beneficial coping mechanism (Schmalbach et al., 2024; Lennartsson et al., 2015). Given this evidence the non-invasiveness and practicality of collecting salivary cortisol, patients subjected to a high acute stressor, such as any form of assault, should be screened for cortisol reactivity. If routinely done, this would allow to retrospectively access it predicted value both for prognosis and therapeutic interventions.

One of the hallmarks of stress disorders is alterations in sleep patterns, with more than 45%–75% of patients showing an anxiety and sleep disorder co-morbidity (Ding et al., 2023; Richards et al., 2020). Can sleep patterns be a prognostic factor for the development of an anxiety disorder after stress exposure? Studies looking at their interrelationship have found that anxiety symptoms and sleep disturbances can independently affect the regulation of the HPA axis, cortisol release, and sympathetic nervous system activation. Thus, whether anxiety or sleep alterations manifest first, both contribute to an increased arousal, increase inflammation, and HPA dysregulation, contributing to the aggravation of both pathologies (Mao et al., 2024). Given their intricate relationship, monitoring sleep patterns is highly relevant for assessing therapeutic efficacy and also relapse, but it can hardly be used to predict resilience to stress exposure (Drake et al., 2014; Kalmbach et al., 2018; Reffi et al., 2023).

Considering the interindividual factors that strongly determine stress resilience, sex emerges as a decisive factor. Differences in sex hormones, estrogen and progesterone in females, and testosterone in males, lead to distinct amygdala reactivity, HPA axis regulation, and stress coping strategies (Ravi et al., 2019). In women and female rodents, estrogen enhances synaptic plasticity and neural activity in the amygdala, hippocampus, and prefrontal cortex, conferring protection in fear responses and promoting fear extinction (Ferree et al., 2011; Glover et al., 2015; Hartsgrove et al., 2025). Further strengthening the role of sexual differences in stress vulnerability, Montero-López et al. (2018) showed that cortisol levels in women vary throughout the menstrual cycle, and Ralevski et al. (2024) demonstrated that progesterone treatment had an anxiolytic effect by reducing cortisol response. Consistently, if in the luteal phase of the menstrual cycle, characterized by elevated progesterone levels and low estrogen, individuals showed an increase in intrusive recollections following stress exposure, strongly contributing to the generalization of fear (Hsu et al., 2021). In men, testosterone appears to attenuate HPA axis reactivity, contributing to generally lower or more controlled cortisol responses compared to females (Ravi et al., 2019). Notably, Rubinow et al. (2005) found that testosterone administration resulted in lower cortisol responses despite elevated ACTH levels following CRH stimulation, suggesting reduced adrenal sensitivity to ACTH and a modulatory effect of testosterone on HPA axis activity. Given this, in males, cortisol responses are lower and less oscillatory, whereas in females, stress leads to higher oscillations in cortisol levels depending on the stage of the hormonal cycle. These hormonal differences might explain why women are generally more susceptible to anxiety, placing them at a heightened risk for the onset of anxiety disorders during their lifespan (Pigott, 1999). Taken together, sex is clearly a risk factor, with women showing an increased susceptibility to the development of anxiety disorders upon stress exposure.

As stated above, the impact of stress is not restricted to brain activity. HPA and the sympathetic system activation have a profound effect on peripheral response, namely in the immune system. Chronic stress leads to an impairment in innate and adaptive immunity (Sarjan and Yajurvedi, 2018) with reduced leukocyte counts, impaired neutrophil function, and increased apoptosis of stem cells and leukocytes induced by dysfunctional levels of corticosterone (Sarjan and Yajurvedi, 2018). Although cortisol has anti-inflammatory properties in acute stress, chronically elevated cortisol levels lead to cortisol resistance, resulting in a reduced ability to suppress the production of pro-inflammatory cytokines, such as IL-6 and TNF-α (Miller et al., 2002). This contributes to chronic inflammation, increasing autoimmune disorders (Skopouli and Katsiougiannis, 2018), depression (Dantzer et al., 2011; Dhabhar, 2014) and anxiety disorders (Passos et al., 2015). Indeed, Ambrée et al. (2018) demonstrated that when exposed to chronic stress, susceptible mice exhibited elevated inflammatory monocytes (Ly6C^++^), increased maturation of splenic dendritic cells, and enhanced brain infiltration of CCR2^+^ immune cells, which correlated with higher basal corticosterone levels. In contrast, resilient mice maintained a more balanced immune profile, characterized by attenuated inflammatory activation. Thus, peripheral biomarkers of inflammation and immune dysregulation positively correlate with the development of a generalized anxiety phenotype. Several brain areas contribute, directly and indirectly, to this link between stress reactivity and immune regulation. As stated above, amygdala reactivity modulates the HPA axis, which in turn modulates cytokine production. Additionally, neurons in the central nucleus of the amygdala (CeA) modulate splenic nerve activity, regulating plasma cell mobilization after immunization. Moreover, several components of the peripheral nervous system bridge the brain and the immune system. Fibers from the sympathetic nervous system (SNS) are central neuronal mediators of stress response, reaching all immune organs and the pre-ganglionic cholinergic innervation of the adrenal gland. Interestingly, fibers from the vagus nerve are involved in dampening inflammation and resolving the acute response to stress exposure (Pavlov and Tracey, 2017). This reflex inhibitory arc represents an additional factor contributing to individual resilience and an opportunity to mitigate the progression to an anxiety disorder.

On the other hand, cytokines can feed back the peripheral response to the brain. Cytokines released in the periphery pass through the blood-brain barrier and activate perivascular macrophages to release their own cytokines (Ravi et al., 2021). In the brain, cytokines can alter brain activity by modulating neurotransmitter metabolism and release (Zipp et al., 2023). Elevated inflammation can influence the activity of the basolateral amygdala (BLA), enhancing fear and anxiety responses (Adkins et al., 2023). This bidirectional interaction between inflammation and amygdala activity forms a positive feedback loop, dysregulates the HPA axis, leads to increased cortisol release, and promotes neuroinflammation in the hippocampus (Lei et al., 2025). Given all this evidence, can the individual variability in the inflammatory response upon stress predict individual susceptibility/resilience? Although this link has not been explored comprehensively, in the Netherlands Study of Depression and Anxiety (NESDA), baseline inflammatory markers and lipopolysaccharide-induced cytokine secretion were increased in patients with anxiety and depressive symptoms and strongly correlated with anxiety severity (Strawn and Levine, 2020; Van Der Feltz-Cornelis et al., 2020). In a second study, treatment of anxiety symptoms led to a decrease in TNF-α, and elevated concentrations of TNF-α, interleukin (IL)-6, and IL-1b were associated with the response to fluoxetine (Amitai et al., 2016). Taken together, cortisol responsiveness together with cytokine production are potential biomarkers for determining whether individuals will develop an anxiety disorder upon exposure to a high acute stressor. In the next section, we will explore the potential role of the endocannabinoid system as an integrator of central and peripheral responses and discuss the hypothesis that endocannabinoid function can be used as a biomarker of stress susceptibility/resilience.

## The endocannabinoid system is a central and peripheral integrator of stress responses

**FIGURE 2:**
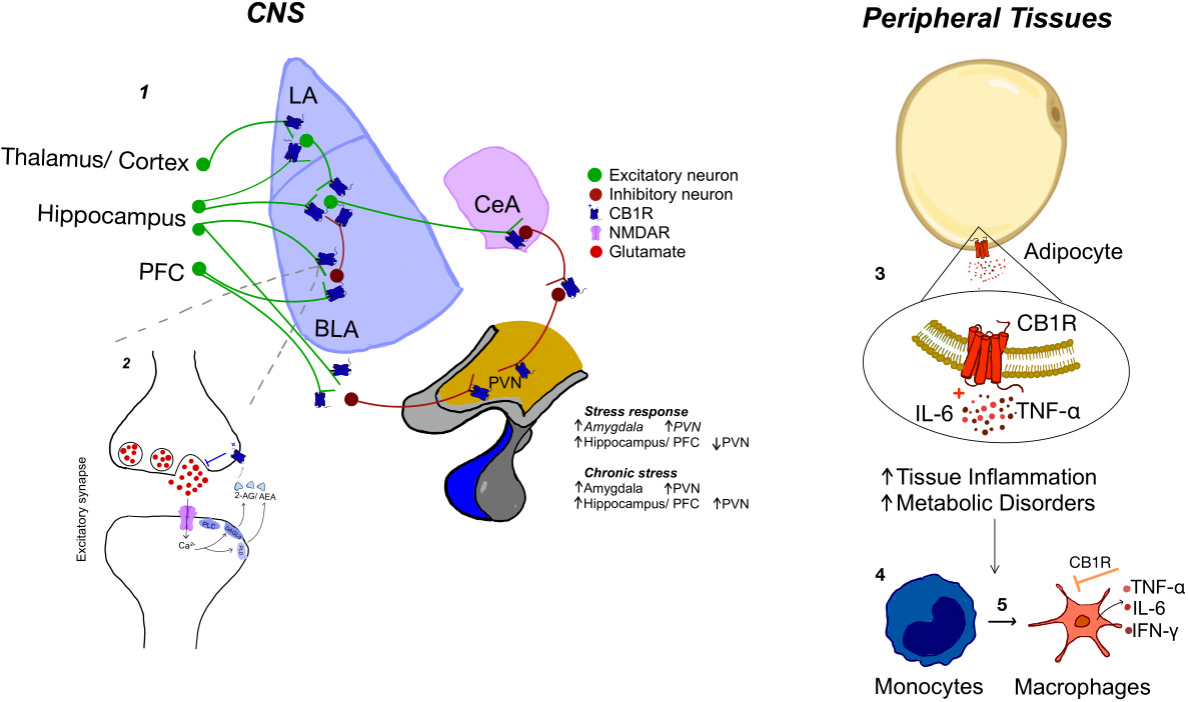
Modulation of the stress response by CB1 receptor activation. **1**: Excitatory projections (green) from the thalamus, cortex, hippocampus, and prefrontal cortex modulate lateral amygdala (LA) and basolateral amygdala (BLA) activation, modulating its output through central amygdala (CeA). Increase in amygdala activation leads to an increase in PVN neurons (hypothalamus) by decreasing inhibition whereas PFC and hippocampus activation leads to a reduction in PVN activation. **2**: CB1R are present in both excitatory and inhibitory pre-synaptic terminals leading to a reduction in neurotransmitter release. Depending on the balance between excitation and inhibition CB1R activation can lead to an increase or decrease in PVN activation. **3**: In the adipose tissue, CB1R hyperactivation triggers adipocytes to release pro-inflammatory cytokines, **4**: which recruit monocytes. **5**: Monocytes differentiate into pro-inflammatory macrophages, further amplifying the release of pro-inflammatory cytokines, contributing to an enhanced inflammatory environment.

The endocannabinoid system regulates synaptic transmission via retrograde signaling and plays a crucial role in restoring homeostasis following stress exposure (Hill et al., 2010; Hillard, 2014). This system comprises cannabinoid receptors CB1 and CB2, their endogenous ligands anandamide (AEA) and 2-arachidonoylglycerol (2-AG), and the enzymes responsible for their respective degradation, fatty acid amide hydrolase (FAAH) and monoacylglycerol lipase (MAGL) (Castillo et al., 2012). Degradation of endocannabinoids (eCBs) ensures that receptor activity is regulated and prevents excessive or prolonged endocannabinoid signaling. CB1 receptors (CB1R) are highly expressed in the central nervous system, including the amygdala, hippocampus and PFC, whereas CB2 receptors (CB2R) are predominantly located in peripheral tissues, being expressed at lower levels in hippocampal neurons (Figure 2; Gong et al., 2006). Activation of CB1R can have an anxiolytic or anxiogenic effect depending on the profile of release of eCBs and previous activation of the neuronal network. If, on one hand, activation of CB1R decreases glutamate release in the amygdala, leading to reduced excitability and attenuation of fear responses (Fonseca et al., 2021), excessive or chronic activation of CB1R can lead to a paradoxical increase in amygdala reactivity due to a decrease in GABA release (Netzahualcoyotzi et al., 2021). This strongly suggests that the variations in the expression or function of CB1Rs are risk factors contributing to the development of anxiety-like behaviors (Haller et al., 2002; Litvin et al., 2013). Additionally, reduced availability of endogenous ligands, in particular AEA, can also contribute to stress-related behaviors by leaving CB1 receptors unbound and inactive. This mechanism has been observed in individuals with PTSD, who often exhibit lower circulating levels of AEA, alongside an upregulation of CB1 receptor availability, potentially reflecting a compensatory response to deficient endocannabinoid signaling (Neumeister et al., 2015). Interestingly, the relationship between CB1R and cortisol is bidirectional, and both are influenced by stress exposure. CB1R are expressed in all structures of the HPA, ranging from the hypothalamus, pituitary, and the adrenal gland (Hillard et al., 2016). As for other structures modulated by CB1R, low levels of eCBs tend to inhibit the HPA response, decreasing cortisol, whereas high levels tend to exacerbate HPA responsiveness, increasing circulating cortisol (Steiner and Wotjak, 2008). Cortisol, on the other hand, increases the availability of eCBs by reducing FAAH activity or, with prolonged exposure, its expression (Gray et al., 2016; Herman et al., 2003). Increasing eCBs may contribute to the negative feedback loop of cortisol on HPA activation (Di et al., 2003; Evanson et al., 2010), which is also consistent with the anxiolytic effect of eCBs, and it constitutes an important component of the coping strategies to repeated stress exposure (Hill and McEwen, 2009; Hill et al., 2009, 2010; Manzanares et al., 1999). This bi-directional regulation of eCBs and cortisol is also seen in the amygdala strengthening their role as key regulators of stress response (Morena et al., 2016; Loomba and Patel, 2025).

Consistent with a general homeostatic role, eCBs also play a crucial role in regulating immunity, influencing both innate and adaptive immune mechanisms (Chiurchiù et al., 2015). Although best known for their role in the central nervous system (Hill et al., 2007), CB1R are also expressed in peripheral tissues, including adipose tissue and the adrenal glands, as well as in several immune cell types including B and T lymphocytes, NK cells, monocytes, neutrophils, and macrophages supporting the idea CB1R acts as a regulatory mechanism limiting excessive immune activation (Figure 2; Nong et al., 2001; Ruiz de Azua et al., 2017; Galiègue et al., 1995). Moreover, pro-inflammatory cytokines such as IL-1β, IL-6, and TNF-α have been shown to regulate CB1 receptor expression in peripheral immune cells (Jean-Gilles et al., 2015), suggesting that under inflammatory conditions, CB1 expression may increase as part of an adaptive mechanism to modulate the immune response. Although the basal expression of CB1 in resting T lymphocytes is relatively low, Börner et al. (2007) demonstrated that T-cell activation via CD3/CD28 stimulation induces a significant upregulation of CB1 expression, highlighting its potential role in adaptive immune regulation. Given the neuro-immune loop described above, individual variations in the eCBs system can have a severe impact on the multiple levels regulating stress responsiveness and long-term coping mechanisms.

Indeed, a recent study identified two single-nucleotide polymorphisms (SNPs - rs12720071 and rs806368) in the *CNR1* gene, which codes for the CB1R, and observed an association of such SNPs with an increased vulnerability to anxiety disorders, particularly among women (Peiró et al., 2020). Moreover, SNPs rs1406977 of *CNR1* lead to a decrease in CB1R mRNA transcripts with a negative impact on its function (Colizzi et al., 2015). Additionally, other SNPs compromise the stability of the CB1R mRNA (Chakrabarti et al., 2006). Polymorphisms in the *FAAH* gene have also been observed, resulting in a reduction of FAAH expression (Dincheva et al., 2015). Extensively studied polymorphism, the rs324420 (C385A), results in the replacement of a proline with a threonine, which reduces enzymatic activity, resulting in an increase in the availability of eCBs, namely AEA. Consistent with a relevant role in stress resilience, women with PTSD who carry the A allele exhibited better physiological and cognitive responses during fear acquisition and extinction tasks (Crombie et al., 2022). This suggests that the *FAAH* polymorphism may influence not only the intensity of stress responses, but also the cognitive strategies individuals use to regulate emotional responses to threat.

## Conclusion and future directions

In this review, we reviewed how stress affects multiple systems, including the nervous, endocrine, and immune systems, and that interactions among the HPA axis, the endocannabinoid system, and the immune system are critical for maintaining stress-response homeostasis. Resilience and vulnerability to stress are further influenced by sex, encompassing hormonal, immune, and neuroanatomical differences, as well as genetic and epigenetic alterations, the latter of which may be transmitted across generations. As such, interindividual variations in endocannabinoid signaling may reflect differences in resilience versus vulnerability to stress. Being at the cross-road of central and peripheral nervous system responses, variation if the eCBs can also account for variation in cortisol response as well as inflammatory cytokine profile. Thus, the combinatorial analysis of eCBs genetic variation with cortisol and cytokine profile offers a promising approach to improve early diagnosis, monitor disease progression, and guide personalized treatment strategies.
